# Myopathie associée au VIH: à propos d’un cas

**DOI:** 10.11604/pamj.2026.53.161.48107

**Published:** 2026-04-16

**Authors:** Soumah Cheick Ousmane, Barry Souleymane Djigué, Touré Mohamed Lamine, Diallo Mohamed Tafsir, Sall Alhassane, Doré Malé, Cissé Fodé Abass

**Affiliations:** 1 Service de Neurologie, Centre Hospitalier et Universitaire Ignace-Deen, Conakry, Guinée,; 2 Clinique Neurologique de Simbaya, Conakry, Guinée

**Keywords:** Myopathie, VIH, syndrome myogène, électromyogramme, cas Clinique, Myopathy, HIV, myogenic syndrome, electromyography, case report

## Abstract

La myopathie liée à l'infection par le virus de l'immunodéficience humaine (VIH) est une complication neuromusculaire fréquente, caractérisée principalement par une atteinte inflammatoire ou toxique des muscles, pouvant entraîner une faiblesse musculaire proximale. Nous rapportons le cas d'un patient séropositif présentant une myopathie confirmée par électromyogramme. Le tableau clinique était dominé par une faiblesse musculaire proximale symétrique, avec asthénie, anorexie, amaigrissement et anémie. La biopsie musculaire n'a pu être réalisée. Ce cas illustre la nécessité d'une reconnaissance clinique et électrophysiologique précoce pour une prise en charge adéquate.

## Abstract (en)

HIV-associated myopathy is a frequent neuromuscular complication characterised mainly by inflammatory or toxic muscle involvement, leading to proximal muscle weakness. We report the case of an HIV-positive patient presenting with myopathy confirmed by electromyography. The clinical presentation was marked by symmetrical proximal muscle weakness, asthenia, anorexia, weight loss, and anaemia. A muscle biopsy was not performed. This case highlights the importance of early clinical and electrophysiological recognition for proper management.

## Introduction

L'infection par le VIH peut entraîner diverses complications systémiques, dont les atteintes musculaires sont particulièrement fréquentes mais souvent sous-diagnostiquées [1]. Parmi celles-ci, la myopathie associée au VIH représente une complication neuromusculaire majeure, pouvant être d'origine inflammatoire, toxique (due aux antirétroviraux) ou liée à l'infection elle-même[2,3].

Cliniquement, la myopathie VIH se manifeste typiquement par une faiblesse musculaire proximale symétrique, une fatigabilité accrue et des douleurs musculaires diffuses [4]. L'électromyogramme (EMG) est un outil clé permettant de confirmer la nature myogène du déficit musculaire, caractérisé par des modifications spécifiques du tracé [5]. Dans ce contexte, la biopsie musculaire reste un examen de référence mais n'est pas toujours réalisable, notamment dans les milieux à ressources limitées [6]. Nous rapportons un cas de myopathie VIH diagnostiqué sur la base de critères cliniques et électromyographiques, soulignant l'importance d'une approche pragmatique dans ce type de contexte.

## Patient et observation

**Information du patient:** nous rapportons le cas d'un patient âgé de 42 ans, suivi pour infection au VIH de type 1 depuis 2019, sous traitement antirétroviral associant ténofovir, lamivudine et dolutégravir. Il a consulté pour l'apparition progressive d'une faiblesse musculaire prédominante aux membres inférieurs, évoluant depuis deux mois.

**Résultats cliniques:** l'examen clinique objectivait un état général conservé, sans signes d'encéphalopathie. Sur le plan moteur, il existait un déficit moteur symétrique des membres inférieurs, prédominant proximalement (côté à 3/5), sans atrophie ni fasciculations. Les réflexes ostéotendineux étaient diminués aux membres inférieurs, normaux ailleurs. Aucune atteinte sensitive ni sphinctérienne n'était rapportée. L'examen des autres appareils était sans particularité.

**Chronologie:** i) 2019: diagnostic d'infection au VIH1 et mise sous traitement ARV; ii) avril 2024: début progressif d'une faiblesse des membres inférieurs; iii) juin 2024: aggravation du déficit moteur, consultation en neurologie; iv) juillet 2024: hospitalisation pour explorations diagnostiques.

**Démarche diagnostique:** un bilan biologique retrouvait une élévation modérée de la créatine phosphokinase (CPK) (520 UI/L). La numération formule sanguine, les enzymes hépatiques, les ions sériques et la fonction rénale étaient normaux. La charge virale du VIH était indétectable, et le taux de CD4 était de 640 cellules/mm^3^. L'imagerie par résonance magnétique médullaire était normale. L'électroneuromyogramme objectivait un tracé myogène avec des potentiels d'unité motrice de faible amplitude et de durée courte. La ponction lombaire montrait un liquide clair, normocytaire, avec une protéinorachie normale. Une biopsie musculaire n'a pas été réalisée faute de moyens techniques.

**Intervention thérapeutique:** une corticothérapie orale à base de prednisone (1 mg/kg/j) a été introduite, associée à une kinésithérapie motrice régulière, sans modification du traitement antirétroviral initial.

**Suivi et résultats des interventions thérapeutiques:** l'évolution a été marquée par une amélioration progressive de la force musculaire, avec un score moteur passé de 3/5 à 4+/5 au bout de six semaines. Le patient a repris une autonomie à la marche avec un appui unilatéral. Aucun effet secondaire notable des corticoïdes n'a été observé durant cette période.

**Perspectives du patient:** le patient s'est montré satisfait de la prise en charge, notamment de la récupération motrice et de l'accompagnement multidisciplinaire. Il est actuellement suivi en consultation externe avec maintien de la kinésithérapie.

**Consentement éclairé:** le patient a donné son consentement éclairé pour la publication de ce cas, dans le respect de l'anonymat et des règles éthiques en vigueur.

## Discussion

La myopathie associée au VIH est une manifestation fréquente mais souvent méconnue, avec une prévalence estimée entre 5 et 30% selon les études [7]. Elle peut être liée à l'action directe du virus, à une réponse immunitaire anormale ou à une toxicité médicamenteuse, en particulier des antirétroviraux [5,8].

Le tableau clinique typique inclut une faiblesse musculaire proximale, des myalgies et une fatigabilité musculaire. L'absence de signes neurologiques permet de suspecter une atteinte purement musculaire [3,4].

L'élévation des CPK est variable et parfois modérée [9]. L'électromyogramme reste un outil de diagnostic essentiel. En contexte de ressources limitées, son apport est précieux, en l'absence de biopsie musculaire. La prise en charge repose sur l'optimisation du traitement antirétroviral, la rééducation et le soutien nutritionnel. Le pronostic dépend de la précocité du diagnostic et de la gestion des facteurs aggravants [10].

## Conclusion

Le diagnostic de myopathie liée au VIH repose sur une combinaison d'arguments cliniques, biologiques et électrophysiologiques. Sa reconnaissance rapide permet une adaptation thérapeutique visant à limiter la progression du déficit. Dans les pays à ressources limitées, l'EMG peut suffire à poser le diagnostic en l'absence de biopsie musculaire.
